# Supplementary material for: Volcanic evolution of an ultraslow-spreading ridge
Source: Nat Commun. 2023 Jul 12;14:4134. doi: 10.1038/s41467-023-39925-0 (PMC10338544; doi:10.1038/s41467-023-39925-0)
Supplement: Supplementary file 1 — Supplementary Information [file 41467_2023_39925_MOESM1_ESM.pdf]

Volcanic evolution of an ultraslow-spreading ridge

H. H. Stubseid*, A. Bjerga, H. Haflidason, L.E.R. Pedersen and R. B. Pedersen

Center for Deep Sea Research and Department of Earth Science, University of Bergen, Allégaten 41, N-5007 Bergen, Norway

Corresponding author. E-mail:* havard.stubseid@uib.no

Supplementary Information

High-resolution bathymetry and sub-bottom profiler

High-resolution bathymetry was collected using AUV mounted Kongsberg EM 2040 multibeam echosounder at 200 kHz with a ping rate of 10 Hz. The beam angle was set to 45/45 giving coverage of 150/150 m. The backscatter db signal was acquired simultaneously as the bathymetry parameter. The AUV had a flight height of ~30-80 meters and a speed of approximately 3.5-4 knots. Onboard data collection and processing were done by Ocean Infinity. We used the Fledermaus software to grid and visualize all the maps with a resolution of 1 m.

Sub-bottom profiler data were acquired with the AUV-mounted source instrument EdgeTech 2205 using EdgeTech Commander version 8.7.3. Data was collected as parallel lines with a spacing of 200-500 m (Fig. S1) together with a centerline perpendicular to the other lines. The SBP sample interval was set to 40 microseconds and the instrument used a 20 ms 1-9 kHz swept-frequency (chirp) source pulse giving a theoretical vertical resolution of 15-25 cm. This instrument has a typical penetration of 15 m in course sands and up to 150 m in clays. During data collection, 100-180 m of vertical data were logged, limiting the resolution of the upper meters of sediment cover. Therefore, based on careful evaluation of the SBP data and measuring of internal reflectors observed within the seismic lines, we estimate a true resolution of 20-30 cm.

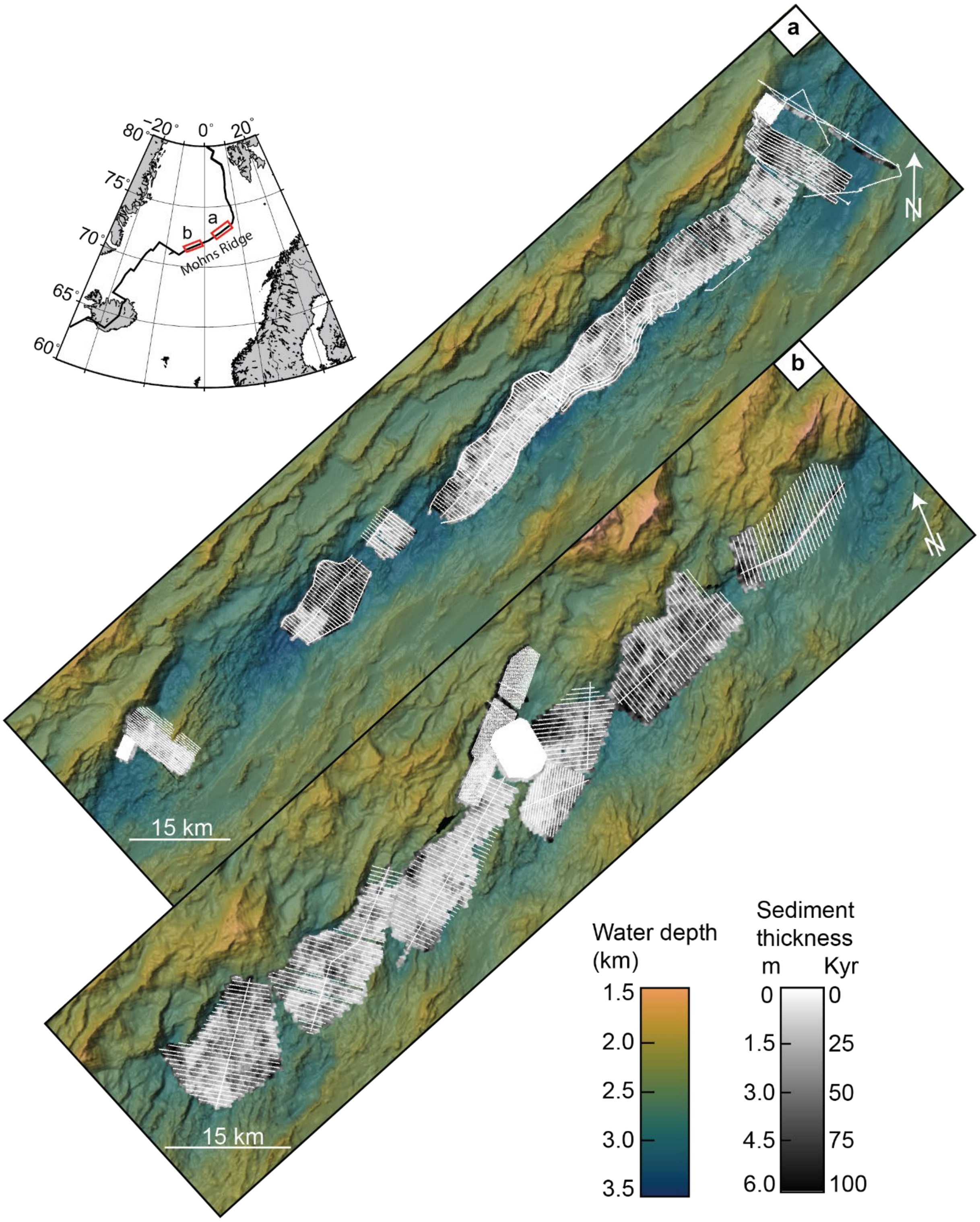

Supplementary Fig. S1: Overview of collected SBP data with draped AUV track lines. Processed and gridded SBP data draped on top of 70-m resolution bathymetry as a grey shade. All AUV track lines are draped on top of the isopach map where **a.** is the northern zone and **b.** is the southern one. Track lines outside the isopach maps are lines that were not completed or did not collect SBP data due to technical issues.

Processing of raw data was done using the Chesapeake Technology software SonarWiz 7. No filters were applied, and we used the envelope signal from the JSF format. A velocity of 1500 m/s was used to convert from two-way travel time to depth. All data were corrected for navigation using updated navigation files extracted from Eiva navigation software. Further, post-processing was done in SonarWiz such as aggregation of files, bottom-tracking, blanking of water-column, and applying of auto gain functions with some manual correction to highlight the sediments. Top basalt reflectors were manually drawn on each line representing the base of the sediment cover. Only areas with visible sediments in the seismic lines were interpreted. The acoustic character is dominated by medium to high-amplitude acoustically laminated sediments draped over the underlying topography. Where the sediment cover is thin, the sediments appear more transparent and discontinuous (Fig. S2). Areas with no sediments (or too little to be seen in the data) represent approximately 20% of the data set.

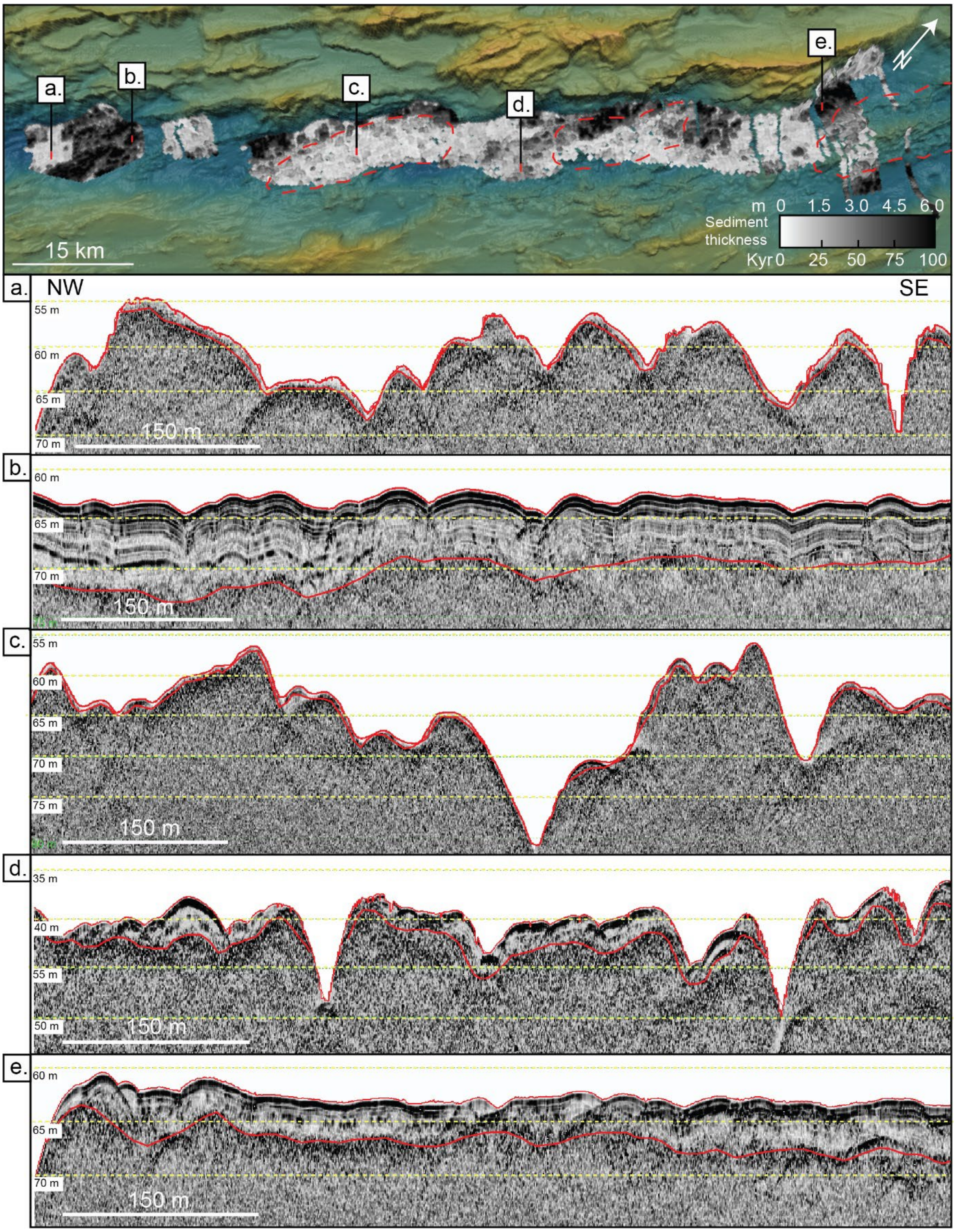

Supplementary Fig. S2: Examples of seismic profiles from various locations within the rift valley. The figure shows 5 selected seismic profiles (A-E) from south to north along the northern segment of the Mohns Ridge. Their location is marked on the isopach map in the upper part of the figure. The different profiles have been selected to represent different settings within the rift valley and areas with varying degrees of sedimentation. There is a 5-meter thickness difference between each dashed yellow line on all the seismic profiles.

Data analysis and areal calculations

All interpretations of backscatter and sub-bottom data were done on top of high-resolution bathymetry for better control of the terrain and locations. Sediment thicknesses were calculated using the “compute thickness” function in SonarWiz, between the manually interpreted top-basalt reflector and the digitized and manually corrected bottom track, with a down-sampling interval of 5 on each reflector. To assess the uncertainty of the calculated sediment thicknesses we considered the resolution of chirp lines and variations in basement pick and bottom tracking. Despite a theoretical resolution of 6-10 cm, we estimated the visual resolution of the chirp lines to be 20-30 cm by measuring individual reflectors. The basement pick appears unaffected by the topography and instead seems to depend more on the thickness of the sediment cover. In areas with thin sediments, with no well-defined stratigraphy, the transition between basaltic basement and sediments is more diffuse. Locally, the bottom track has some minor variations that could influence the calculated sediment thicknesses. Therefore, based on these assessments and any errors related to the manual interpretation of the seismic data, we estimate an uncertainty of 0.5 m (~8 Kyr) in the calculated sediment thickness. The sediment thicknesses were exported as XYZ (latitude, longitude, and sediment thickness) text files and gridded to an isopach map in the Fledermaus software using the default weighted moving average grid function with a weight diameter of 3 and a cell size of 175 m, giving the average sediment thickness for an area of 0.03 km^2. The cell size was selected to close the spacing between the lines that were up to 500 m without any further interpolation. A final gridded surface was draped onto the underlying bathymetry for visualization of the sediment distribution (Fig. S3). The accuracy of the gridding was assessed by acquiring direct measurements of sediment thicknesses from SBP data, correlated with the length of actual cores, and compared with backscatter and bathymetric maps using the 3D function of Fledermaus (Figs. S6, S7 & S8).

We find a near-perfect match between the backscatter maps, thickness measurements, core lengths, and the isopach map, verifying the accuracy of the gridded sediment thicknesses.

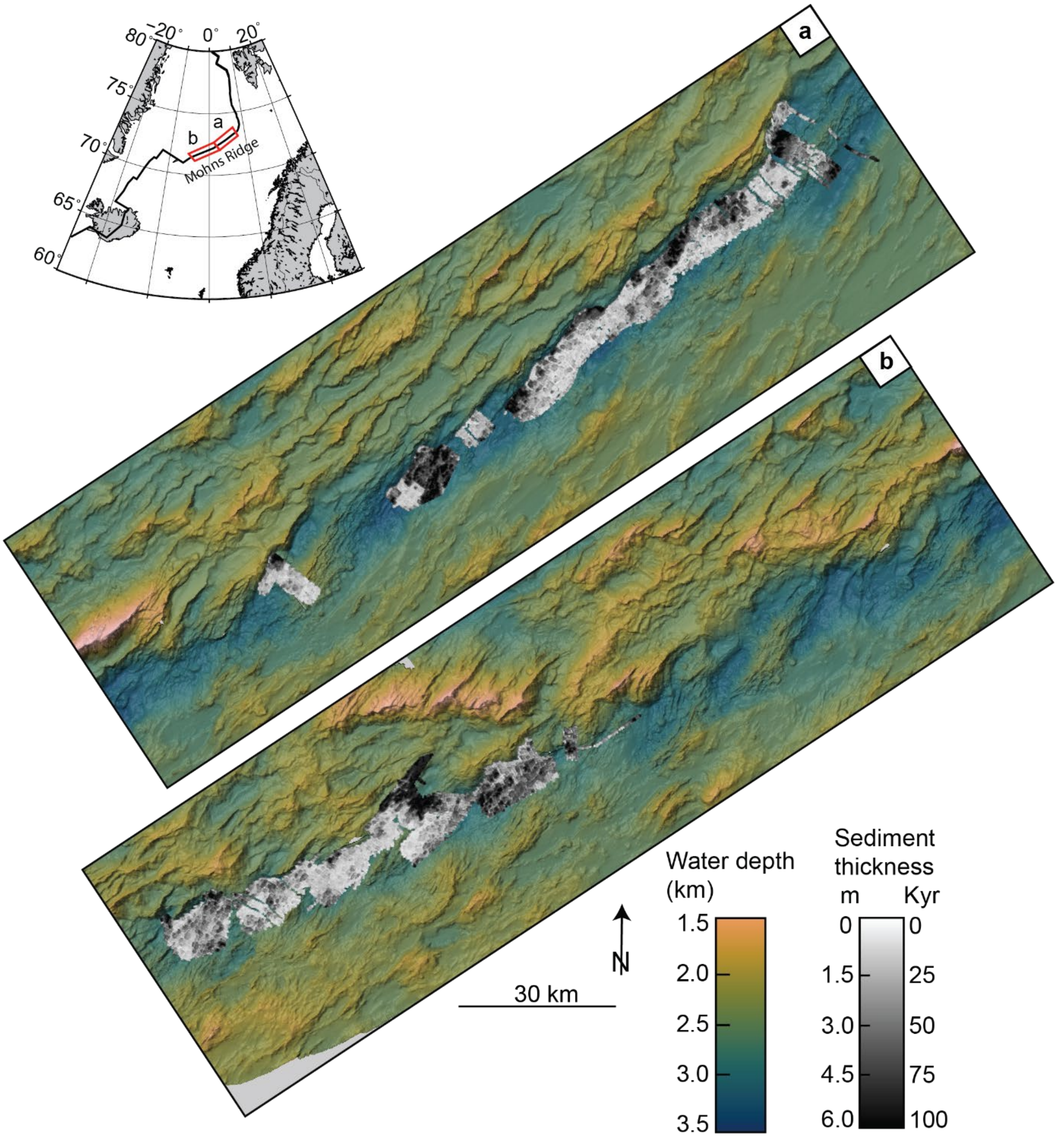

Supplementary Fig. S3: Overview of the main part of the Mohns Ridge showing the entire isopach map. a. The northern part of the isopach map, with sediment the thickness draped onto 70 m resolution bathymetry. Note that the three southernmost surveyed areas within this figure are missing from Fig. 1 in the main text. Therefore, all data is included here. **b.** shows the southernmost part of the isopach map. All collected high-resolution bathymetry and back-scatter data cover the same area as for the sediment thicknesses.

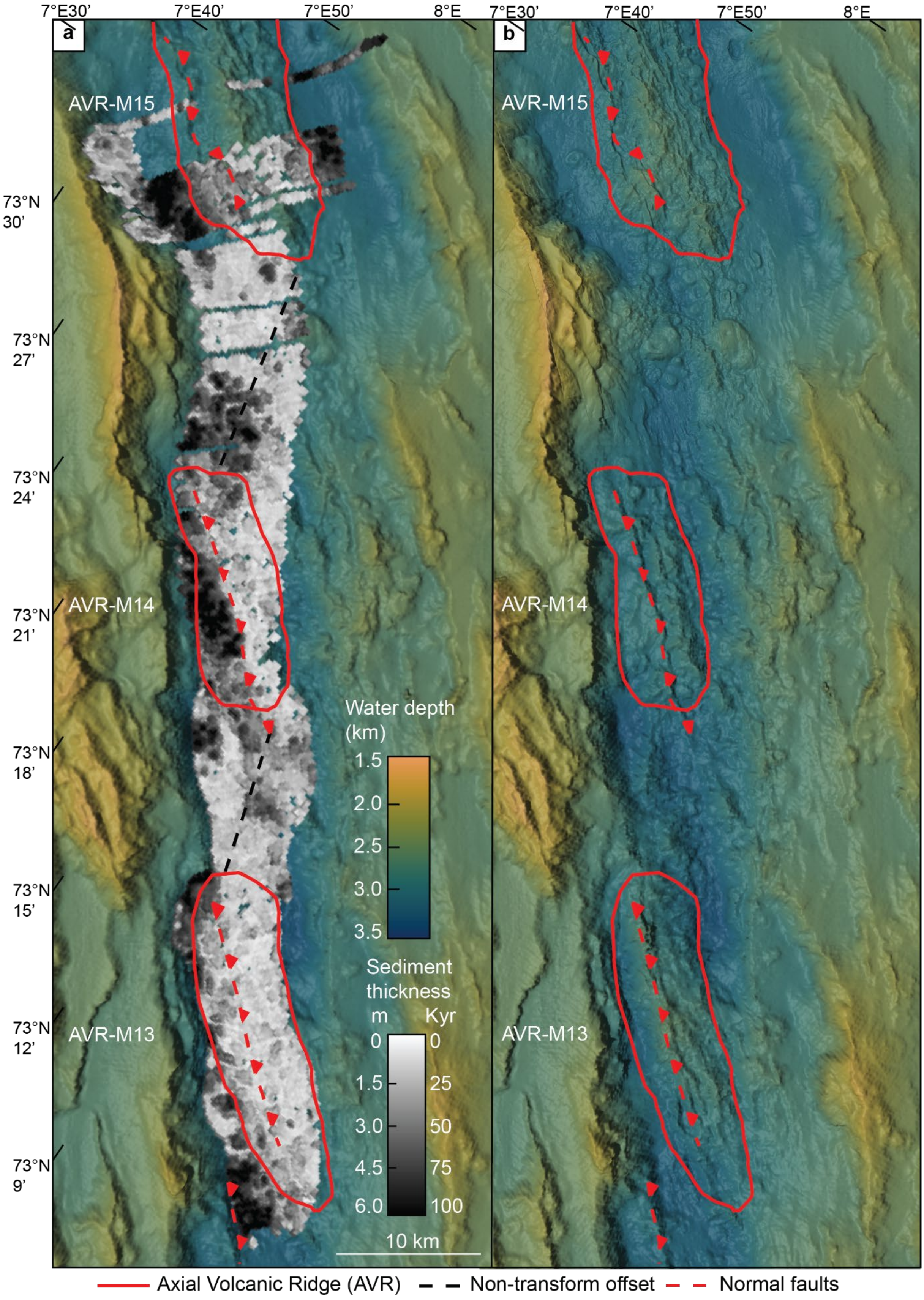

Supplementary Fig. S4: Overview of the isopach map and its underlying bathymetry from the northern part of the Mohns Ridge. Clean 70-m resolution (25-m resolution around AVR-M15) bathymetry is added to the isopach map to enable interpretation of the underlying morphologies of the rift valley. This figure corresponds to Fig. 1a in the main text.

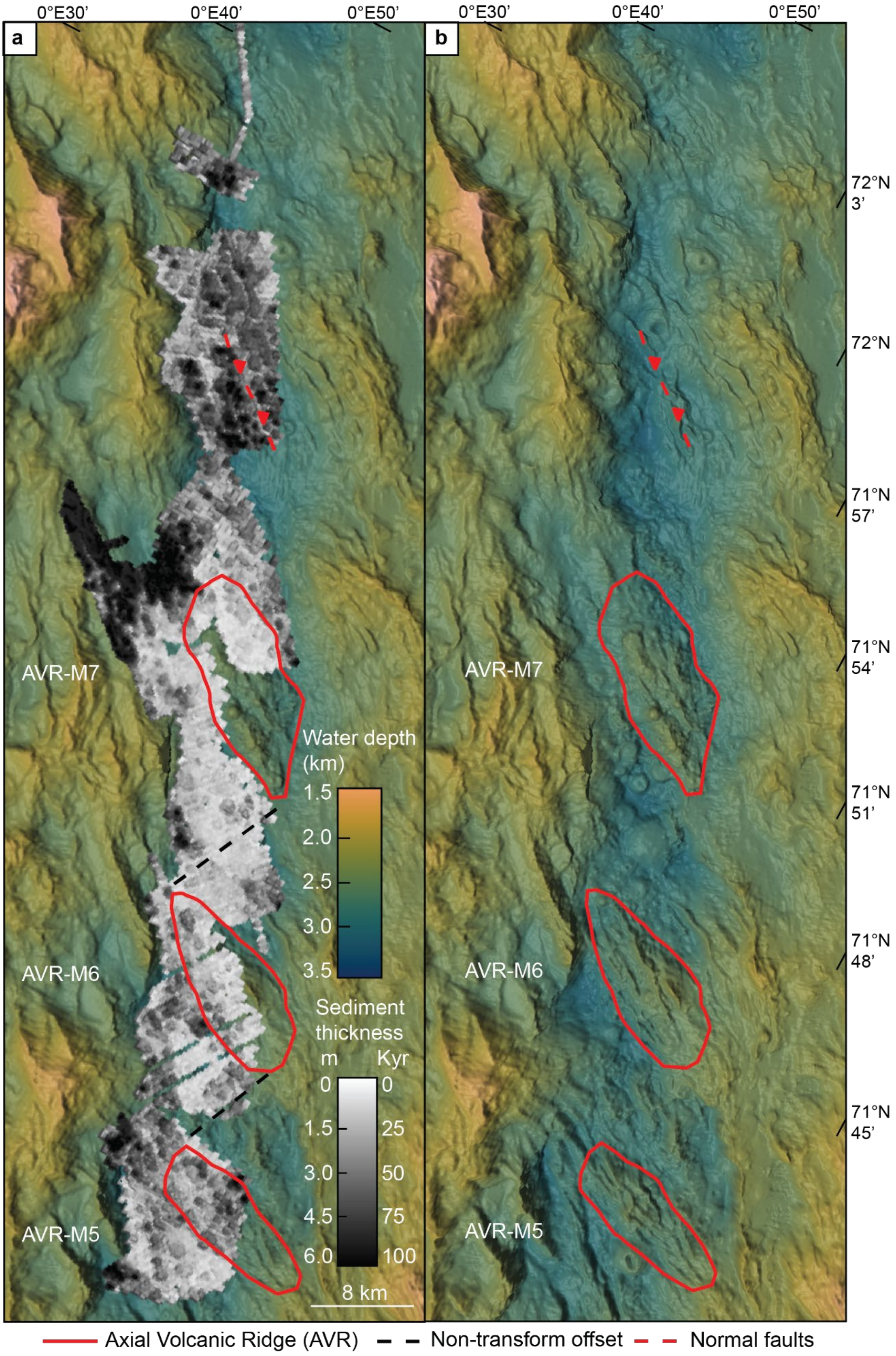

Supplementary Fig. S5: Overview of the isopach map and its underlying bathymetry from the southern part of the Mohns Ridge. Clean 70-m resolution bathymetry is added to the isopach map to enable interpretation of the underlying morphologies of the rift valley. This figure corresponds to Fig. 1b in the main text.

Comprehensive data analysis was performed on all thickness measurements directly from the chirp profiles. In total, we obtained ~1.06 million thickness measurements from the entire surveyed area comprising 467 survey lines. Areas with no sediments, or too little to be seen in the data (<50 cm), contain no thickness measurements. We used Python to assess the total area without thickness measurements. All lines were assessed individually and individual measurements within a line were sorted geographically by Easting due to some survey lines having unsorted data. This rendered survey lines that had a lot of bending in the Easting axis unusable for further data analysis and removed, leaving the dataset with 954511 thickness measurements from 431 survey lines. Although the median length between two sediment thickness measurements never passes 4 m, any distance between two sediment thickness measurement that exceeds 25 m are regarded as a "hole". The length of each hole was assessed by measuring the distance between the start and stop coordinates for individual holes. The total hole distance of the entire dataset accounts for around 20% of the total line distances. To include the holes as part of the thickness statistics, all the holes were filled with dummy points with a 0 m sediment thickness value. To assign a suitable number of dummy points for each hole, the median distance between the actual thickness measurements from each individual survey line was used to set the distance between dummy points. After filling all the holes, we end up with a total of ~1.21 million data points of which ~0.3 million are dummy points. As a quality control, all dummy points were visualized together with real thickness measurements to make sure they plot correctly.

To further characterize the sediment thickness data, each point was correlated to a co-located slope value. The slope was calculated from AUV DTM rasters at 1m resolution projected in WGS84 UTM 31N using the native GDAL slope analysis tool in QGIS 3.24.1 to produce a slope raster in degrees. The slope data was associated with each sediment point using the QGIS Sample Raster Values Tool and added to each point in a separate column. To avoid steep slopes

where sediments tend to not accumulate, we excluded all thickness measurements and dummy points in areas steeper than 30 degrees. These steep areas represent regions of thin sediment not because of young volcanism but rather as an effect of topography. After slope consideration, 25% of the data point was removed and we end up with a total of 0.91 million points further used for data analysis and sediment statistics.

The sediment thickness dataset was analyzed, and all measurements were visualized in a boxplot with bins for each 1 meter (see Fig. 3 in the main text). We argue that all remaining thickness measurements after corrections are the result of hemipelagic sedimentation, and the thickness is, therefore, a representative proxy for the age of the underlying lavas. The data distribution follows an exponential decrease from thin sediments towards thicker ones with a "half-life" calculated using the best-fit line function on the data bins.

To analyze the sediment distribution separately for AVRs and areas outside AVRs (see Fig. 3a and b in the main text), the outline of individual AVRs was manually drawn as polygons in QGIS. The polygons were then used to define a Boolean 'inside' or 'outside' value for each thickness measurement defined by a QGIS area select for all points within the polygons which was then inverted to define the 'outside' points. We find that 35% of the datapoint are within AVRs whereas 65% are outside. These two areas were then analyzed and visualized separately, following the same steps as above, to study the sediment distribution and volcanic age pattern of these different terrains.

Sediment cores and radiocarbon dating

A comprehensive coring campaign was conducted to establish and evaluate the sedimentation rates for the entire Mohns Ridge. A total of 21 gravity cores were collected during cruises with RV G.O Sars in 2020 and 2021 (Fig. 2 in main text and Supplementary Table S1). Only one core, GS20-230-19GC, retrieved no sediments. Gravity cores were collected using a 5 m steel

tube hitting the seafloor at a speed of approximately 1 m per second. Cores were cut into sections, labeled, and placed in cold storage. Further processing was conducted at the EarthLab, Department of Earth Science at the University of Bergen. Every core section was split into two halves and scanned on the ITRAX XRF core scanner [1] combining X-ray reflectance (XRF), radiographic X-ray imaging, and optical imaging (RGB, 3x3000 pictures). The element composition was measured every 2nd mm.

Supplementary Table S1: Overview of sediment cores. The table includes map ID, core ID, latitude, longitude, core length, and water depth for all the collected sediment cores.

Map ID	Core ID	Latitude	Longitude	Core Length (cm)	Water depth (m)
1	GS20-230-03GC	72° 17.6913' N	01° 40.8273' E	308	2650
2	GS20-230-06GC	72° 29.6665' N	02° 30.4937' E	197	3000
3	GS20-230-07GC	72° 44.9100' N	03° 56.5621' E	80	2500
4	GS20-230-09GC	72° 45.2560' N	03°49.8750' E	62	3050
5	GS20-230-10GC	72° 45.3210' N	03°49.9760' E	129	3026
6	GS20-230-11GC	72° 47.4420' N	03°56.6080' E	313	2490
7	GS20-230-12GC	72° 44.6755' N	04° 18.1880' E	135	2676
8	GS20-230-13GC	72° 55.2010' N	04° 58.8690' E	345	3000
9	GS20-230-14GC	73° 01.2580' N	05° 29.5430' E	272	3000
10	GS20-230-15GC	73° 10.3810' N	06° 12.5630' E	275	3360
11	GS20-230-16GC	73° 13.3730' N	06°32.7790' E	323	3360
12	GS20-230-17GC	73° 16.0380' N	06° 46.9960' E	134	3010
13	GS20-230-18GC	73° 18.7400' N	06° 49.9290' E	385.5	3137
14	GS20-230-19GC	73° 27.2990' N	07° 12.5550' E	0	2764
15	GS20-230-20GC	73° 23.1130' N	07° 19.0510' E	294	2560
16	GS20-230-21GC	73° 20.2150'N	07° 24.9140' E	286	3000

17	GS21-235-GC04	71° 54.7540' N	01° 18.7970' W	349	2770
18	GS21-235-GC05	72° 05.3620' N	00° 07.8560' W	440	3122
19	GS21-235-GC06	72° 44.9210' N	03° 49.5950' E	150	3085
20	GS21-235-GC07	72° 45.3630' N	03° 49.6340' E	70	3010
21	GS21-235-GC08	72° 45.3240' N	03° 49.9790' E	120	3010

Five selected cores were sub-sampled, sieved, and cleaned before specimens of the planktonic foraminifera species N. pachyderma (sin) were handpicked for radiocarbon dating (Figs. S9 and S10). Radiocarbon dating analyzes were performed at the Radiocarbon Dating Laboratory, Department of Geology, Lund University, Sweden. Ages were corrected for total isotopic fractionation and converted to calendar years using the Marine20 calibration curve[2]. The age results were correlated and compared with the elemental record from the remaining 15 cores, providing a robust framework for the age model (Fig. S11). Our results reveal that sedimentation rates vary from 4.1-7.2 cm/Ka with an average of 6 cm/Ka for the rift valley floor of the Mohns Ridge. This is significantly higher than in the open ocean of the Norwegian-Greenland Sea with a sedimentation rate in the order of 2-4 cm/Ka [3,4,5,6].

Several studies have suggested that age-to-depth ratios in sediment cores might not be accurate due to potential disturbance from re-sedimentation and bioturbation [7,8,9]. In high-latitude deep sea areas, the disturbance from bioturbation can be high owing to generally low sedimentation rates. To evaluate the extent and magnitude of vertical mixing in these areas, tephra particles have been applied as a tracer to document the rate and quantity of bioturbation. The vertical distribution of the volcanic glass particles in the studied cores shows that the effective mixing of the sediments does not exceed 2–3 cm and the original layer is not markedly displaced. The amplitude of the original signal is therefore only slightly smoothed[10,11].

Our seismic lines (Fig. S2) show only well-stratified sediments without any evidence of post-depositional disturbance. This is further supported by the well-laminated and stratified sediments recovered in all cores from the rift valley (Figs. S9 and S10), and the lack of disturbance in the depth-to-age ratio of the dated cores. We therefore argue that the deposited sediments result from a steady hemipelagic sedimentation environment. Currently, no measurements of bottom currents along the Mohns Ridge are available to further evaluate small-scale variations and influence by local currents. The only observed bottom current to date is a major southward-going current far-off axis in the Greenland Sea[12] suggesting stable currents within the rift valley. A recent study by Bosse and Fer [13] documents an intermediate current at 1000m depth going northwards along the Mohns Ridge. Even though this current is far up in the water column, potentially without any major effects on the bottom currents, this could have an influence leading to the slight increase in sedimentation rate from south to north along the ridge. The northern part of the Mohns Ridge is also closer to sedimentary sources from the Norwegian-Svalbard continental margin e.g.[14].

Our isopach map report the average sediment thickness for an area of 0.03 km^2 and small-scale variations may therefore be masked. We see no systematic pattern between water depth or seafloor morphology that may affect the sedimentation rate, except for slopes above 30° that rarely contain any well-defined sediments. Our isopach map shows several examples of deep basins with and without thick sediments, as well as ridges and volcanic cones with varying degrees of sediment cover. These observations conclude that the measured sediment thickness is a result of hemipelagic sedimentation without local topographic effects.

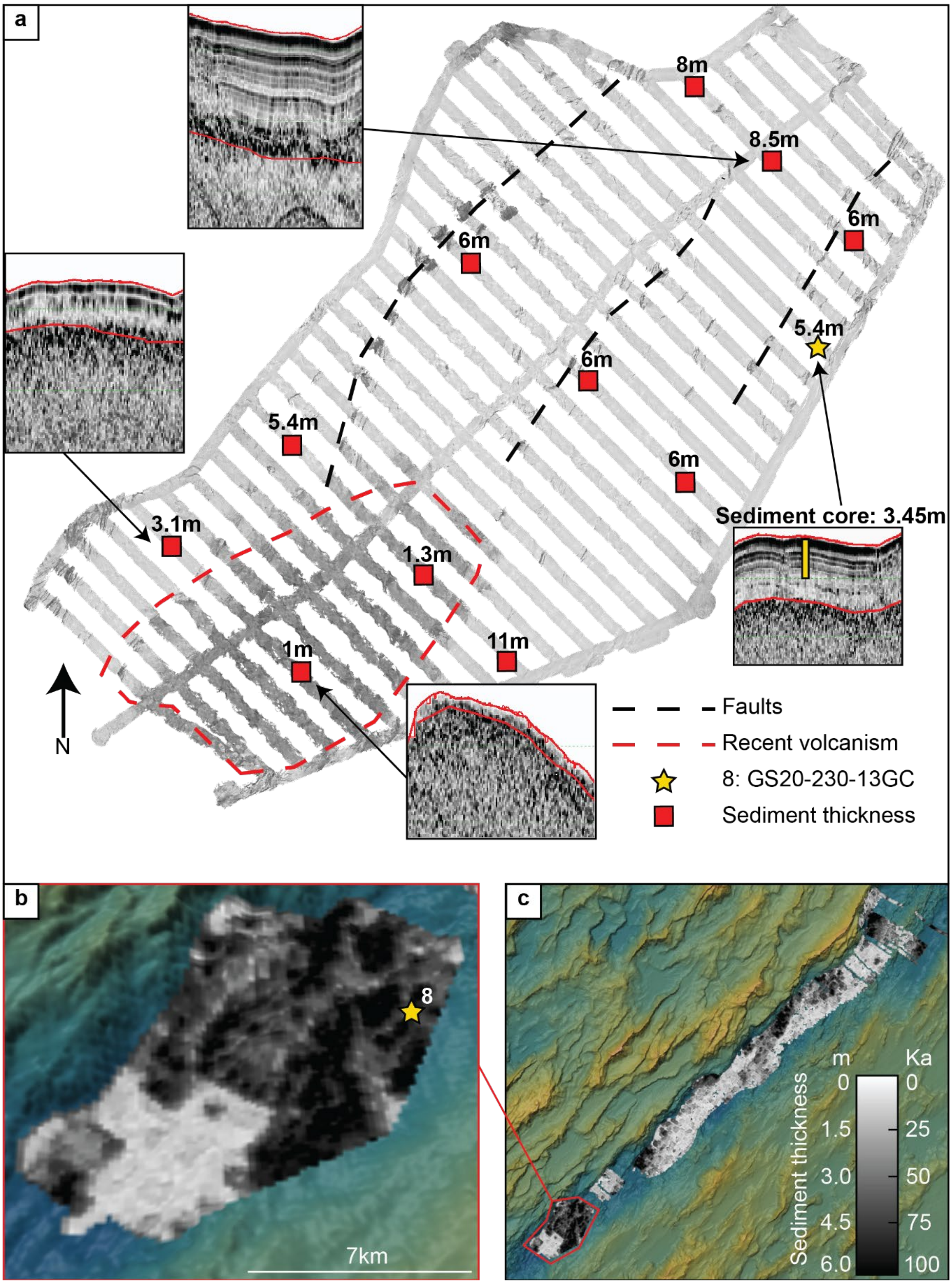

Supplementary Fig. S6: Sediment thicknesses and backscatter map for the area of core 8. a. Backscatter map (AUV data) of 1m resolution is shown to further confirm the degree of sedimentation. Accurate measurements of the sediment thicknesses, acquired from the sub-bottom profiles, are shown as red squares in various locations. Insets of the seismic lines are used to visualize the sedimentary deposits. GS20-230-13GC is marked as a yellow star with its exact length presented within the seismic profile for the sampled area. **b.** The figure shows the sediment distribution and age of the volcanic crust for a sub-area of the northern zone. **c.** Overview of the northern part of the isopach map. The detailed sub-area is marked with red lines.

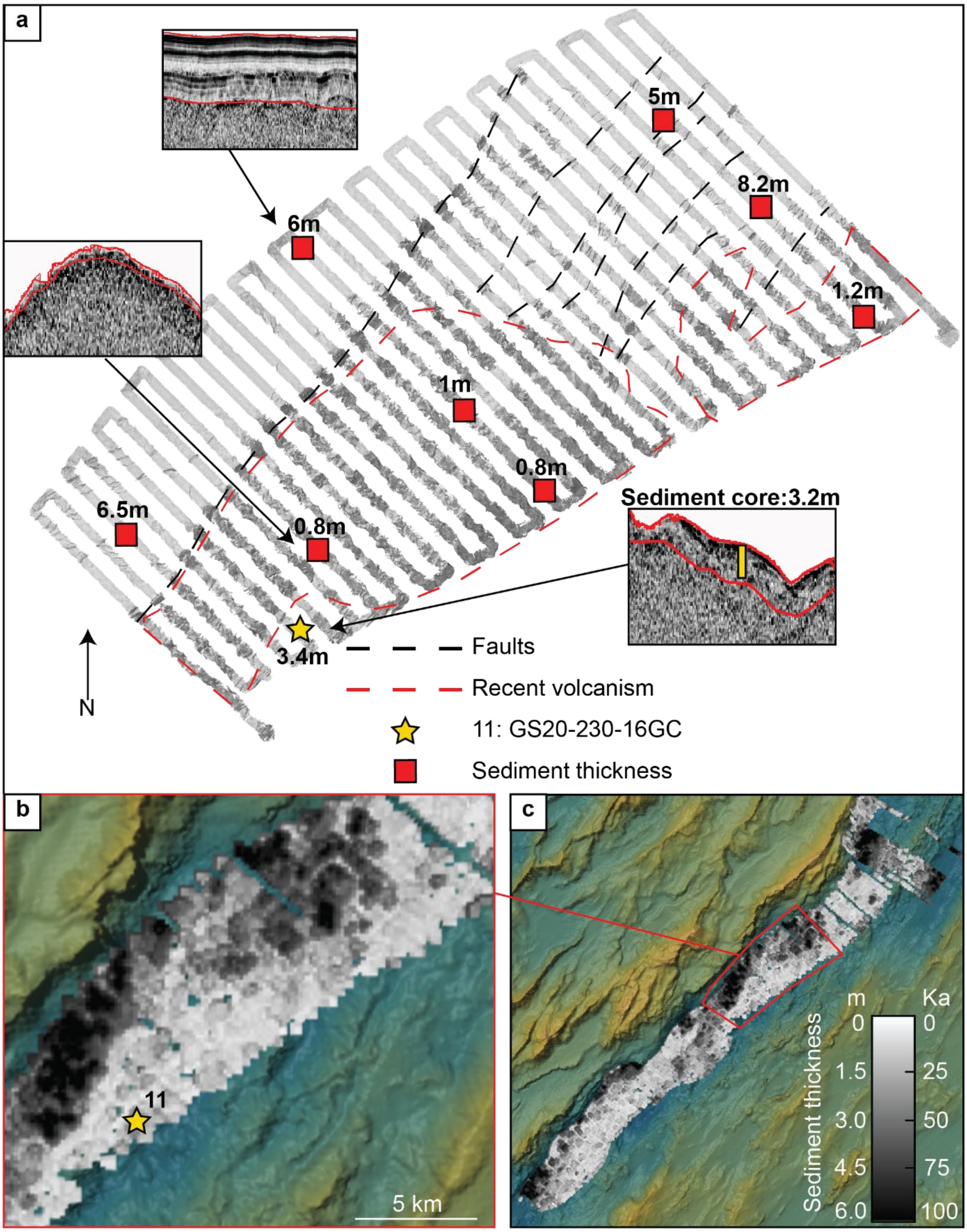

Supplementary Fig. S7: Sediment thicknesses and backscatter map for the area of core 11. a. Backscatter map (AUV data) of 1m resolution is shown to further confirm the degree of sedimentation. Accurate measurements of the sediment thicknesses, acquired from the sub-bottom profiles, are shown as red squares in various locations. Insets of the seismic lines are used to visualize the sedimentary deposits. GS20-230-16GC is marked as a yellow star with its exact length presented within the seismic profile for the sampled area. **b.** The figure shows the sediment distribution and age of the volcanic crust for a sub-area of the northern zone. **c.** Overview of the northern part of the isopach map. The detailed sub-area is marked with red lines.

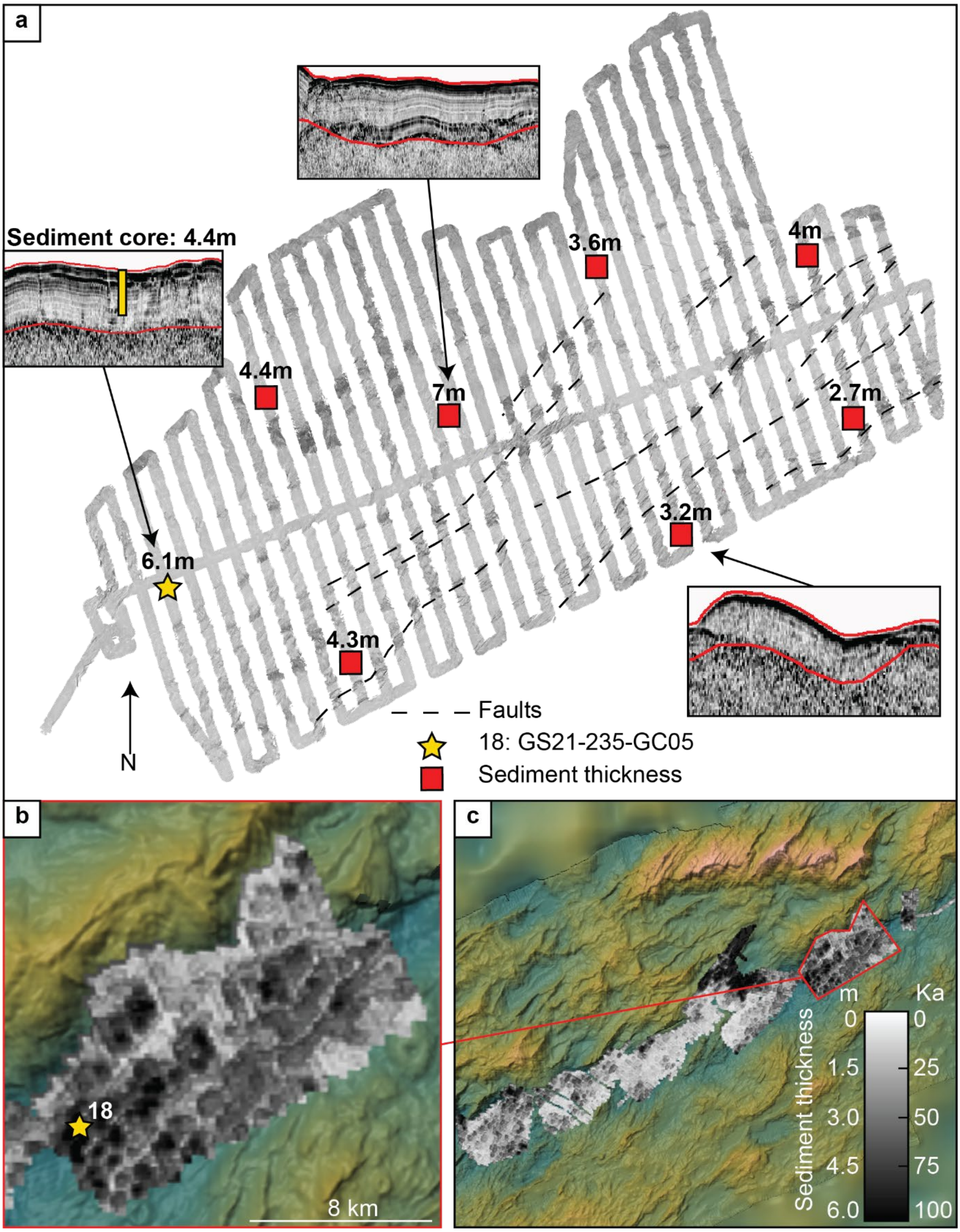

Supplementary Fig. S8: Sediment thicknesses and backscatter map for the area of core 18. a. Backscatter map (AUV data) of 1m resolution is shown to further confirm the degree of sedimentation. Accurate measurements of the sediment thicknesses, acquired from the sub-bottom profiles, are shown as red squares in various locations. Insets of the seismic lines are used to visualize the sedimentary deposits. GS21-235-GC05 is marked as a yellow star with its exact length presented within the seismic profile for the sampled area. **b.** The figure shows the sediment distribution and age of the volcanic crust for a sub-area of the northern zone. **c.** Overview of the northern part of the isopach map. The detailed sub-area is marked with red lines.

Supplementary Table 2: Overview of marine AMS ^{14}C-dates of the 5 dated cores. The ^{14}C dates are calibrated using the Marine20 calibration model (Heaton et al., 2020).

Map ID.	Core No.	Lab. ID	Depth in core (cm)	Material	^{14}C (BP)	^{14}C ± 1δ	Cal. BP (1δ) Median	Median (interval)
1	GS20-230-03GC	LuS 17211	11.0 - 12.0	*N. pach* (sin)	9 350	50	**10 000**	(9898 -10116)
		LuS 17212	31.0 - 32.0	*N. pach* (sin)	14 380	100	**16 580**	(16398-16751)
		LuS 17213	51.0 - 52.0	*N. pach* (sin)	18 990	120	**22 070**	(21920-22240)
		LuS 17214	71.0 - 72.0	*N. pach* (sin)	25 290	210	**28 655**	(28425-28915)
		LuS 17426	110.5 - 111.5	*N. pach* (sin)	44 600	+1200/-1000	**46 485**	(45109-47560)
		LuS 17427	165.0 - 166.0	*N. pach* (sin)	36 500	400	**40 535**	(40195-40906)
		LuS 17428	209.0 - 210.0	*N. pach* (sin)	44 700	+1200/-1000	**46 465**	(45365-47395)
8	GS20-230-13GC	LuS 17215	55.0 - 56.0	*N. pach* (sin)	9 035	50	**9 550**	(9462-9633)
		LuS 17216	100.0 - 101.0	*N. pach* (sin)	17 350	120	**20 055**	(19872-20241)
		LuS 17477	155.5 - 156.5	*N. pach* (sin)	29 700	200	**33 375**	(33116-33640)
		LuS 17478	235.0 - 236.0	*N. pach* (sin)	40 700	+1000/-900	**43 255**	(42475-43878)
11	GS20-230-16GC	LuS 17205	15.0 - 16.0	*N. pach* (sin)	3 390	35	**3 075**	(2985-3164)
		LuS 17206	30.0 - 31.0	*N. pach* (sin)	6 500	40	**6 770**	(6677-6857)
		LuS 17207	46.0 - 47.0	*N. pach* (sin)	8 615	55	**9 075**	(8979-9183)
		LuS 17208	55.0 - 56.0	*N. pach* (sin)	9 120	45	**9 650**	(9538-9731)
		LuS 17209	106.0 - 107.0	*N. pach* (sin)	16 430	100	**18 940**	(18790-19079)
		LuS 17210	157.0 - 158.0	*N. pach* (sin)	24 440	190	**27 745**	(27514-27951)
		LuS 17479	225.0 - 225.5	*N. pach* (sin)	30 200	300	**33 885**	(33601-34216)
		LuS 17480	272.0 - 273.0	*N. pach* (sin)	40 700	+1000/-900	**43 255**	(42475-43876)
16	GS20-230-21GC	Beta-647321	140.5 - 141.5	*N. pach* (sin)	15 570	50	**17 990**	(17828-18153)
		Beta-647322	209.5 - 210.5	*N. pach* (sin)	25 370	100	**28 745**	(28606-28886)
18	GS21-235-GC05	LuS 17483	168.0 - 169.0	*N. pach* (sin)	15 920	70	**18 410**	(18272-18544)
		LuS 17484	286.0 - 287.0	*N. pach* (sin)	35 600	500	**39 790**	(39325-40232)

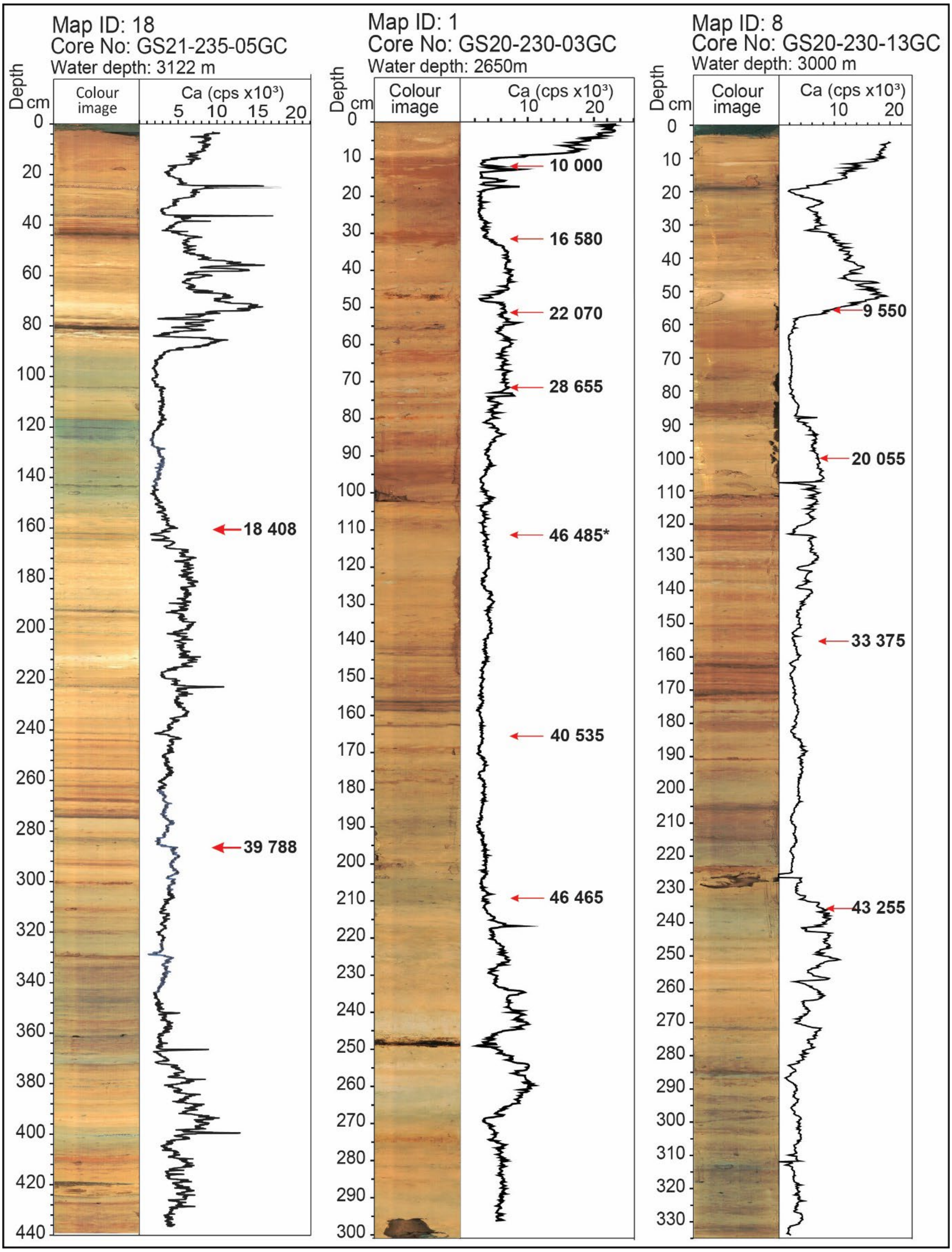

Supplementary Fig. S9: Overview of 3 dated cores. The figure shows the color image of three of the dated cores, from south to north: nr. 18 (GS21-235-05GC), nr. 1 (GS20-230-13GC) and nr. 8 (GS20-230-13GC). Note that core nr. 1 was over penetrated and the top part of the core is missing. The Ca curve, measured every 2nd mm using the XRF element scanner, is presented together with the acquired ^{14}C dates for each core (details in Table S2). Note that core nr 1 exhibit one old-over-younger age (46 485*). This is outside the age trend for the rest of the core, and an isolated data point at this age is not reliable as it is on the limit of the method.

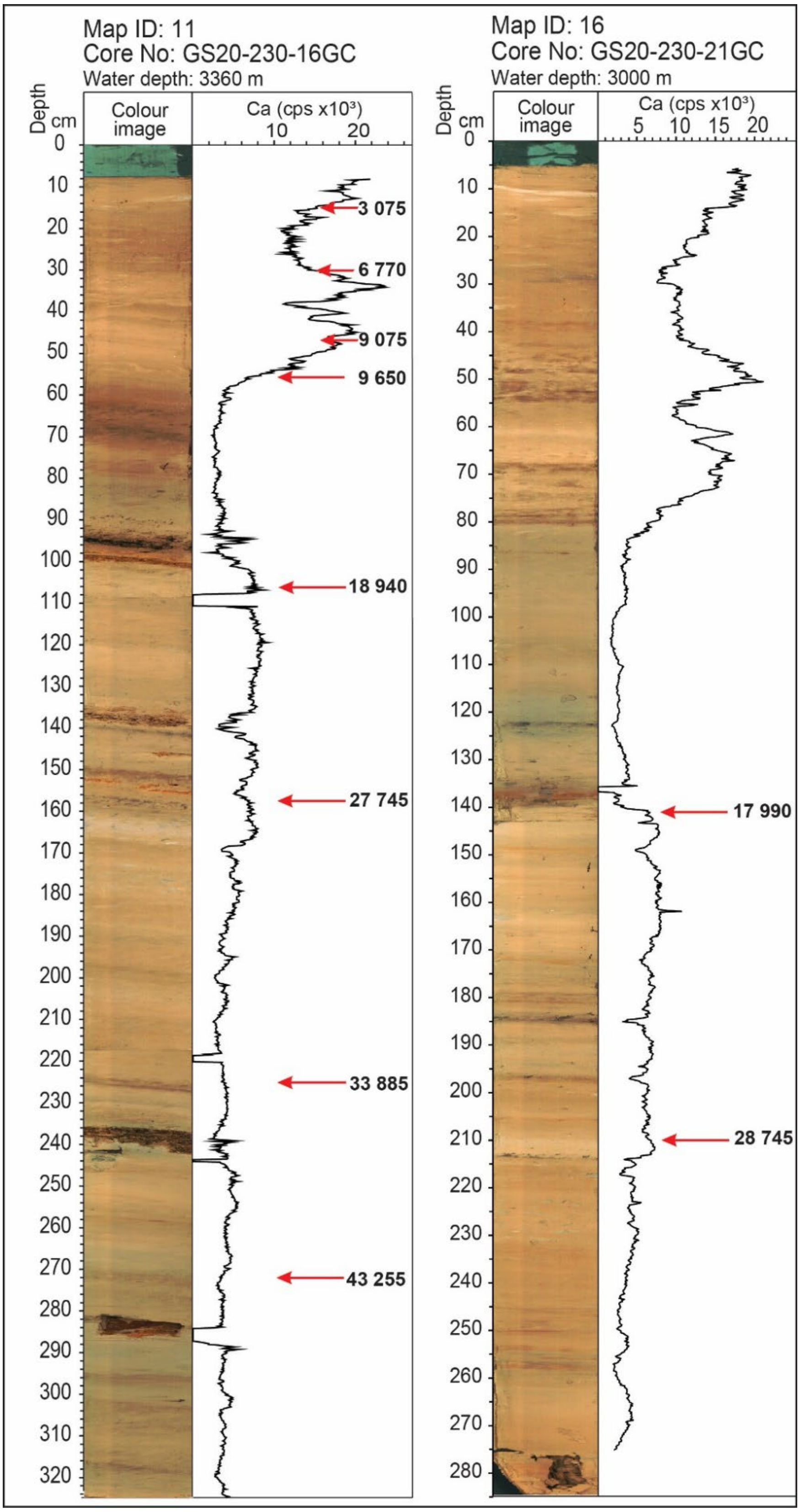

Supplementary Fig. S10: Overview of 2 northernmost dated cores. The figure shows the color image of the two northernmost dated cores: nr. 11 (GS20-230-16GC) and nr. 16 (GS20-230-21GC). The Ca curve, measured every 2nd mm using the XRF element scanner, is presented together with the acquired ^{14}C dates for each core (details in Table S2).

The Ca geochemical composition (semi-quantitative) was determined by the ITRAX XRF core scanning system at a spatial resolution of 2 mm, using a molybdenum X-ray tube at the EarthLab core scanning facility, at the University of Bergen. The Ca-counts represent the concentration of carbon preserved in the marine core stratigraphy where the variability reflects the productivity in the surface open ocean from calcareous planktons like planktonic foraminifera and coccoliths and the variability of the regional paleo oceanographic conditions[15, 16, 17]. Therefore, the Ca curve is used for correlation between the different cores (Fig. S11) and to establish the sedimentation rate by comparing the chemo stratigraphy and using ^{14}C ages from core nr. 1, 8, 11, 16, and 18.

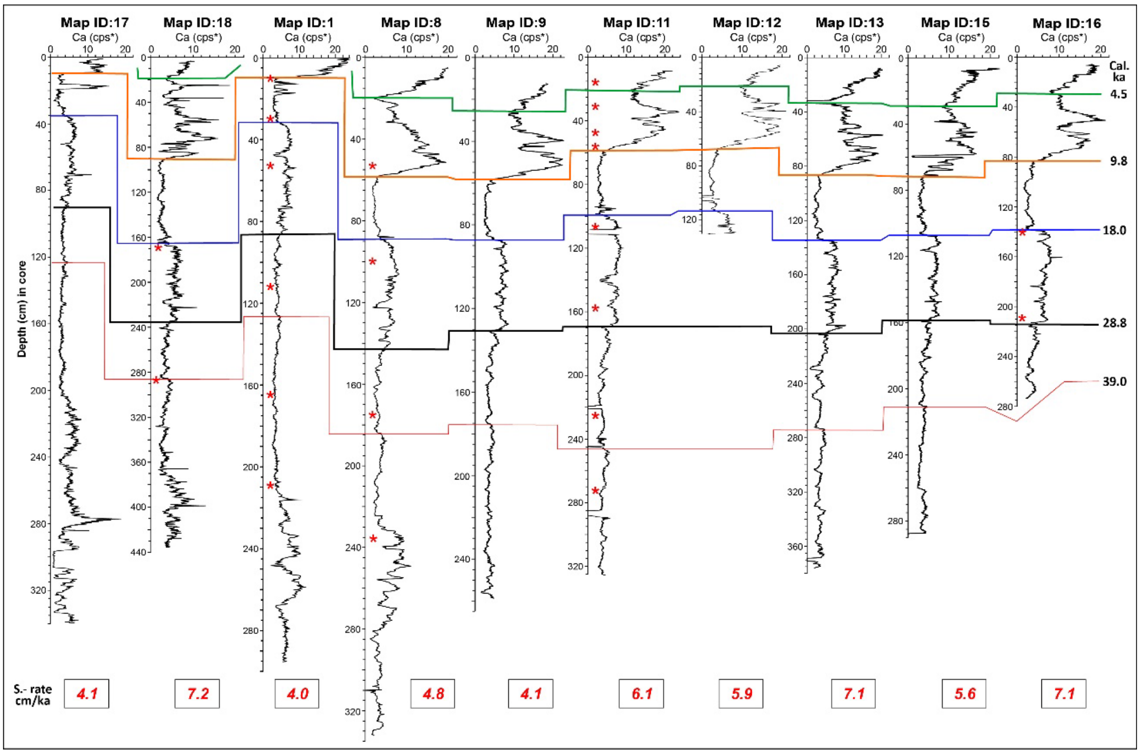

Supplementary Fig. S11: Compilation of Ca-curve for 10 of the studied cores. The figure shows the XRF element scanning of Ca for 10 of the studied cores. The cores are sorted geographically from south to north along the ridge and the average sedimentation rate is reported as red numbers for each core. Here, anomalies in the Ca curve that are present in all the cores are used to correlate their stratigraphy. ^{14}C ages, presented in Fig. S10, Fig. S11, and Table S2, from the five dated cores (nr. 1, 8, 11, 16, and 18) are used to date the different anomalies and to establish the sedimentation rates for the cores. Red stars mark the location of all dated samples. This further strengthens our age model and verifies an average sedimentation rate of 6cm/Ka for the entire rift valley. Note that the top part of core 17 and 1 is lost due to over-penetration.

References

1. Croudace IW, Rindby A, Rothwell RG. ITRAX: description and evaluation of a new multi-function X-ray core scanner. *Geological Society, London, Special Publications* **267**, 51-63 (2006).

2. Heaton TJ*, et al.* Marine20—the marine radiocarbon age calibration curve (0–55,000 cal BP). *Radiocarbon* **62**, 779-820 (2020).

3. Fronval T, Jansen E. Eemian and early Weichselian (140–60 ka) paleoceanography and paleoclimate in the Nordic seas with comparisons to Holocene conditions. *Paleoceanography* **12**, 443-462 (1997).

4. Fronval T, Jansen E, Haflidason H, Sejrup HP. Variability in surface and deep water conditions in the Nordic seas during the last interglacial period. *Quaternary Science Reviews* **17**, 963-985 (1998).

5. Koç N, Jansen E. Response of the high-latitude Northern Hemisphere to orbital climate forcing: Evidence from the Nordic Seas. *Geology* **22**, 523-526 (1994).

6. Sarnthein M*, et al.* Variations in Atlantic surface ocean paleoceanography, 50°-80° N: A time-slice record of the last 30,000 years. *Paleoceanography* **10**, 1063-1094 (1995).

7. Clague DA*, et al.* Geologic history of the summit of Axial Seamount, Juan de Fuca Ridge. *Geochemistry, Geophysics, Geosystems* **14**, 4403-4443 (2013).

8. Portner RA, Clague DA, Helo C, Dreyer BM, Paduan JB. Contrasting styles of deep-marine pyroclastic eruptions revealed from Axial Seamount push core records. *Earth and Planetary Science Letters* **423**, 219-231 (2015).

9. Zuhr AM*, et al.* Age-Heterogeneity in Marine Sediments Revealed by Three-Dimensional High-Resolution Radiocarbon Measurements. *Frontiers in Earth Science* **10**, (2022).

10. Haflidason H, Eiriksson J, Kreveld SV. The tephrochronology of Iceland and the North Atlantic region during the Middle and Late Quaternary: a review. *Journal of Quaternary Science: Published for the Quaternary Research Association* **15**, 3-22 (2000).

11. Sjøholm J, Sejrup HP, Furnes H. Quaternary volcanic ash zones on the Iceland Plateau, southern Norwegian Sea. *Journal of Quaternary Science* **6**, 159-173 (1991).

12. Hansen B, Østerhus S. North atlantic–nordic seas exchanges. *Progress in oceanography* **45**, 109-208 (2000).

13. Bosse A, Fer I. Mean structure and seasonality of the Norwegian Atlantic Front Current along the Mohn Ridge from repeated glider transects. *Geophysical Research Letters* **46**, 13170-13179 (2019).

14. Bruvoll V, Breivik AJ, Mjelde R, Pedersen RB. Burial of the Mohn-Knipovich seafloor spreading ridge by the Bear Island Fan: Time constraints on tectonic evolution from seismic stratigraphy. *Tectonics* **28**, (2009).

15. Brendryen J, Haflidason H, Sejrup HP. Norwegian Sea tephrostratigraphy of marine isotope stages 4 and 5: prospects and problems for tephrochronology in the North Atlantic region. *Quaternary Science Reviews* **29**, 847-864 (2010).

16. Helmke JP, Bauch HA, Röhl U, Mazaud A. Changes in sedimentation patterns of the Nordic seas region across the mid-Pleistocene. *Marine Geology* **215**, 107-122 (2005).

17. Rothwell RG. Twenty years of XRF core scanning marine sediments: What do geochemical proxies tell us? In: *Micro-XRF studies of sediment cores*). Springer (2015).
